# *Copia *and *Gypsy *retrotransposons activity in sunflower (*Helianthus annuus *L.)

**DOI:** 10.1186/1471-2229-9-150

**Published:** 2009-12-23

**Authors:** Marco Vukich, Tommaso Giordani, Lucia Natali, Andrea Cavallini

**Affiliations:** 1 Dipartimento di Biologia delle Piante Agrarie, Università di Pisa, Via del Borghetto 80, I-56124 Pisa, Italy

## Abstract

**Background:**

Retrotransposons are heterogeneous sequences, widespread in eukaryotic genomes, which refer to the so-called mobile DNA. They resemble retroviruses, both in their structure and for their ability to transpose within the host genome, of which they make up a considerable portion. *Copia*- and *Gypsy*-like retrotransposons are the two main classes of retroelements shown to be ubiquitous in plant genomes. Ideally, the retrotransposons life cycle results in the synthesis of a messenger RNA and then self-encoded proteins to process retrotransposon mRNA in double stranded extra-chromosomal cDNA copies which may integrate in new chromosomal locations.

**Results:**

The RT-PCR and IRAP protocol were applied to detect the presence of *Copia *and *Gypsy *retrotransposon transcripts and of new events of integration in unstressed plants of a sunflower (*Helianthus annuus *L.) selfed line. Results show that in sunflower retrotransposons transcription occurs in all analyzed organs (embryos, leaves, roots, and flowers). In one out of sixty-four individuals analyzed, retrotransposons transcription resulted in the integration of a new element into the genome.

**Conclusion:**

These results indicate that the retrotransposon life cycle is firmly controlled at a post transcriptional level. A possible silencing mechanism is discussed.

## Background

The mobile component of the genome is represented by sequences, called transposable elements (TEs), which are potentially able to change their chromosomal location (transposition) through different mechanisms. This feature has a cladistic significance and TEs are subdivided into two main classes accordingly to their mechanism of transposition, retrotransposons (REs, class I) and DNA transposons (class II). Class I elements, which includes all REs, can transpose through a replicative mechanism which involves the transcription of an RNA intermediate by the enzyme machinery of the host cell, and subsequent retrotranscription to cDNA and integration into the host genome by the enzymes encoded by the retrotransposon RNA. Such a "copy and paste" mechanism has been largely successful during the evolution of eukaryotes in which class I elements represent the largest portion of higher plant genomes. In the case of *Oryza australiensis*, the amplification of retrotransposons doubled the genome size [1].

Retrotransposons are divided into autonomous and non-autonomous elements, according to the presence of ORFs that encode RE enzymes. Non-autonomous elements do not carry enough coding capacity to allow them to transpose autonomously, nevertheless they are able to move using enzymes encoded by other elements [2].

Basically, the genome of autonomous REs is organized in two domains: the *gag *domain, which is committed towards the production of virus like particles, and the *pol *domain, whose encoded enzymes are used for processing RE-mRNA and obtaining a double stranded DNA to be integrated into the genome. The occurrence of long terminal repeats (LTRs) flanking the retrotransposon genome distinguishes REs into two main classes, namely LTR- and non-LTR-retrotransposons. LTRs carry promoter elements, polyadenilation signals and enhancers regulating the transcription of retroelements.

*Gypsy *and *Copia *LTR retrotransposons are two ubiquitous classes [3,4] of plant REs that differ in the order of genes encoded by *pol*. *Gypsy *and *Copia *elements resemble retroviruses in their structure due to the presence of LTRs and internal ORFs. LTR-retrotransposons lacking internal coding domains, such as TRIMs (Terminal-repeat Retrotransposons In Miniature [5]) and LARDs (LArge Retrotransposons Derivatives [6]) have also been described. Formerly discovered in *Solanum tuberosum *and *Arabidopsis*, TRIMs have been reported in monocots and dicots while LARDs, which were shown to be transcribed, have been reported in *Triticeae *[4,6].

Over the last two decades, some examples have correlated the emerging of RE activity in the genome with a stress mediated reaction: Tnt1 and Tto1 in *Nicotiana *and Tos17 in rice showed stress-induced (by tissue culture) transcription and transposition [7-10], while these elements were not transcribed in standard culture conditions.

Large genome sequencing of grass plants showed that REs are responsible for extensive changes in genome structure and, surprisingly, dramatic differences were reported even among individuals belonging to the same species [11,12]. A remarkable example of retrotransposon dynamics as an evolutionary adaptive mechanism within an ecological system is offered by BARE1 elements in wild barley [13].

It has been proposed that REs restructuring action plays a role in regulating gene expression [14,15]. It has been suggested that allelic variation in non-genic (regulatory) sequence may be involved in heterosis, i.e. the superior performance of hybrids in respect of their parents [16]. In this sense, the old epithet of "junk" for such repeated sequences, which have affected genome structure and function, is becoming obsolete.

Though the interplay between REs and host genome has allowed genome expansion and the evolution of the gene expression regulating network, the vast majority of REs seem to be inactivated by a large spectrum of mutations. Only few elements have been shown to transpose autonomously and data from EST libraries in grasses indicate that most are poorly transcribed [17-19]. However, it is conceivable that the activity of REs should be limited by the host genome because of their potential mutagenic action.

The control of TEs activity is related to RNA interference, a process mediated by small RNAs which derive from a number of different precursors, determining chromatin specific methylation and condensation, and RNA degradation [20]. In the fission yeast *Schizosaccharomyces pombe*, a basal level of transcripts matching centromeric DNA repeats is the substrate for the production of small RNAs that maintain heterochromatin structure through histone methylation [21]. A silencing pathway of REs and repetitive sequences, driven by anti-sense small RNAs, is well described in *Drosophila *[22] and in *Arabidopsis *[23].

In plants, retrotransposon dynamics have mainly been investigated in grasses and other monocotyledons, and in dicotyledons such as *Arabidopsis*, *Gossypium *species, *Nicotiana*, and *Lotus japonicus*. Recently, genome expansion related to the amplification of REs has been shown to occur in the evolution of three *Helianthus *hybrid species adapted to extreme environments [24,25].

The cultivated sunflower (*Helianthus annuus*) has a medium-large sized genome (3.30 pg DNA per haploid genome [26]. The occurrence of *Copia *and *Gypsy *REs has been reported a few years ago for the first time [27] and, lately, the main portion of sunflower genome was shown to be composed by REs [28]. The analysis of cpDNA suggested that the *Helianthus *genus originated between 4.75 and 22.7 million years ago while the *Helianthus *extant lineages appeared between 1.7 and 8.2 million years ago [29].

With the aim to study RE activity in a relatively young and medium-large genome sized species, we have analyzed retrotransposons transcription and integration of new REs in plants of cultivated sunflower.

## Results

### REs expression in the *Helianthus annuus *genome

Sunflower repeated sequences were previously isolated from a *Helianthus annuus *partial genomic library by hybridization with labeled genomic DNA [27]. Among these, one *Copia*-like sequence (pHaS211 [EMBL acc. number AJ009967], hereafter called C211) and three *Gypsy*-like sequences (pHaS13 [AJ532592], pHaS22 [FM208278], and pHaS30 [FM208279], hereafter called G13, G22, and G30, respectively) resulted as being medium repeated, with a copy number per haploid genome ranging from 4,000 to 16,000 (Cavallini, unpublished). These sequences were studied with respect to their RNA transcription.

**Figure 1:**
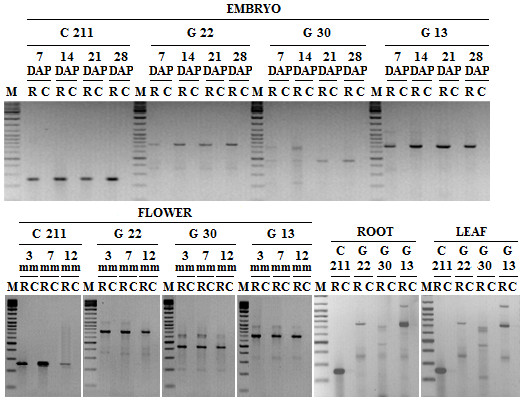
**RT-PCR analysis of sunflower retrotransposons**. RT-PCR products of C211, G22, G30, and G13 REs from total RNAs of embryos at 7, 14, 21, 28 days after pollination (DAP), flowers at three different development stages (3, 7, and 12 mm in length), roots and leaves. For each sample, RT-PCR reactions were performed on retrotranscribed RNA (R) and on the corresponding non-retrotranscribed RNA (C). Molecular weight marker (M, Gene Ruler DNA Ladder Mix, Fermentas) was also loaded.

Specific PCR primers were designed on conserved domains matching the *RNAseH *and the *Integrase *gene of *Copia *and *Gypsy *elements, respectively. RT-PCR experiments were performed to assess the occurrence of RE transcripts in different organs such as root, leaf, flower (at three different stages) and embryo (at four different stages), collected from HCM line plants. For each organ and stage, amplified fragments of the expected length were obtained (Fig. 1), indicating that the retroelement families studied are actively transcribed in all the organs analyzed. Additional PCR products were obtained, that might have been originated by the transcription of retrotransposon remnants and/or related elements.

Three amplified fragments for each RT-PCR product were cloned and sequenced (EMBL acc. numbers FM208268-FM208277). Sequences belonging to the *Copia*-like element did not show sequence polymorphism (Table 1). This may indicate that only one or a few *Copia *REs belonging to C211 family are transcribed, so that polymorphism cannot be detected by sequencing only three PCR products. All nine sequences belonging to *Gypsy*-like REs were different, indicating that different elements of all analyzed *Gypsy*-like families are transcribed.

**Table 1:** Number of sites, nucleotide diversity per site (π) and per non-synonymous and synonymous sites, of coding portions of different retrotransposon families of *H. annuus*.

Sequence	N. of sequences	N. of sites	π	N. of non-synonymous sites	π_a_	N. of synonymous sites	π_s_	π_a_/π_s_
G13	3	710	0.0451	538.50	0.011	169.50	0.154	0.071
G22	3	740	0.1378	573.06	0.041	164.94	0.477	0.086
G30	3	467	0.0828	359.22	0.042	105.78	0.222	0.189
C211	3	252	0.0000	197.17	0.000	54.83	0.000	--

Eight out of the nine *Gypsy *sequences and the *Copia *ones do not show stop codons. In spite of the low number of sequences analyzed, this result indicates that many of the expressed REs encode functional protein sequences. Within the G13, G22, and G30 families, the ratio between synonymous and non synonymous substitutions was 0.071, 0.086, and 0.189, respectively, i.e., close to zero (Table 1). Such low ratios are usually found in coding gene sequences and they indicate conservative selection. A BLAST search on EST databases using sequences of three *Gypsy *and one *Copia *REs as queries, indicated that RE families related to those analyzed in our experiments are transcribed also in other Asteraceae species (Table 2) for which EST data are available, both belonging to the *Helianthus *genus (*H. argophyllus*, *H. ciliaris*, *H. exilis*, *H. paradoxus*, *H. tuberosus*) or to other genera (*Artemisia annua*, *Senecio cambrensis*, *S.chrysanthemifolius, S. vulgaris*, *Carthamus tinctorius*, *Centaurea solstitialis*, *Cichorium intybus*, *Lactuca perennis*, *L. sativa*, *L. serriola*, *Zinnia elegans*).

**Table 2:** Hits showing sequence similarity with G13, G30, G22, and C211 REs in EST libraries of different Asteraceae species.

Subfamily	Tribe	Species	G13			G22			G30			C211		
			T	S	U	T	S	U	T	S	U	T	S	U
Asteroidee	Heliantheae	*Helianthus annuus*	5	1	2	5	1	3	-	-	-	2	1	-
		*H. argophyllus*	1	-	-	3	-	-	-	-	-	-	-	-
		*H. ciliaris*	1	-	-	-	-	-	-	-	-	-	-	-
		*H. exilis*	1	-	-	1	-	-	-	-	-	-	-	-
		*H. paradoxus*	3	-	-	1	-	-	-	-	-	-	-	-
		*H. tuberosus*	2	-	-	2	-	-	-	-	-	-	-	-
		*Zinnia elegans*	2	2	-	5	5	-	1	1	-	-	-	-
	Anthemideae	*Arthemisia annua*	1	-	1	1	-	1	20	-	20	-	-	-
	Senecioneae	*Senecio cambrensis*	1	-	1	1	-	1	-	-	-	1	-	1
		*S. chrysanthemifolius*	1	-	1	-	-	-	-	-	-	-	-	-
		*S. vulgaris*	1	-	1	1	-	1	-	-	-	-	-	-
Carduoideae	Cardueae	*Carthamus tinctorius*	1	-	-	1	-	-	-	-	-	-	-	-
		*Centaurea solstitialis*	-	-	-	1	-	1	-	-	-	-	-	-
Cichorioideae	Cichorieae	*Cichorium intybus*	1	-	1	3	-	3	-	-	-	-	-	-
		*Lactuca perennis*	-	-	-	1	-	-	-	-	-	-	-	-
		*L. sativa*	2	2	-	2	-	-	-	-	-	-	-	-
		*L. serriola*	-	-	-	1	-	-	-	-	-	-	-	-

T: total of EST matches, S: transcripts from stressed tissues, U: transcripts from unstressed tissues. Matches were considered reliable according to their E-value (e^-5^) with variable minimum query coverage: 10% for G13 (710 bp-long) and for G22 (740 bp-long), 45% for G30 (467 bp-long) and for C211 (252 bp-long).

### Isolation and analysis of LTRs

Putative full-length *Gypsy*-like LTRs were isolated by two-step chromosome walking, following the method reported for sunflower *Copia*-like LTRs [30].

**Figure 2:**
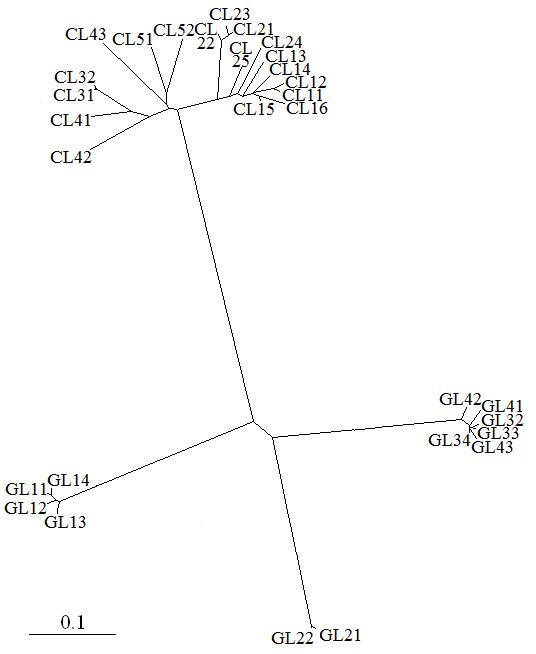
**Phylogenetic analysis of isolated LTRs**. Neighbour-joining analysis of alignment of LTRs of *H. annuus*. Bar represents distance (percent divergence) between all pairs of sequence from the multiple alignments (CL: putative *Copia *LTR; GL: putative *Gypsy *LTR).

Twelve putative *Gypsy *LTRs (EMBL acc. numbers FM177929-FM177940) and 18 putative *Copia *LTR (FM177911 - FM177928[30]) were aligned and a consensus tree, based on nucleotide sequences, was obtained by the neighbor-joining analysis (Fig. 2). The tree showed a clear distinction between *Gypsy *and *Copia *LTRs. Moreover, *Copia *LTRs resulted to be more uniform than *Gypsy *ones (Table 3), for which three distinct families are observed (Fig. 2). Calculation of nucleotide diversity along the relatively uniform *Copia *LTRs indicated that diversity is higher in the central region of LTR than at both ends (not shown).

**Table 3:** Number of sites, number of sites excluding gaps, nucleotide diversity (π) and its sampling variance for *Gypsy *and *Copia *LTR sequences of *H. annuus*.

Sequence	N. of sequences	N. of sites	N. of sites excluding gaps	π	variance of π
*Gypsy *LTRs	12	615	536	0.3842	0.0024
*Copia *LTRs^1^	18	454	353	0.1325	0.0002

^1^Data from Vukich et al. [30]

CLUSTAL alignment clearly showed conserved putative TATA box promoter both in the *Gypsy *(TATAAA) and in the *Copia *(TATATATA) LTRs. To analyze the structure of a putative RE promoter, isolated *Copia *and *Gypsy *LTRs were scanned for *cis*-elements against the PLACE database [31]. In all the LTRs analyzed, stress-responsive *cis*-elements as Myb, Myc, and WRKY motifs were found. *Cis*-elements typical of constitutively expressed genes such as Dof-related elements [32] and CACT boxes [33] were observed. Also many putative light responsive elements, such as GATA boxes and GT1 binding sites [34], tissue-specific motifs such as SEF3 binding sites [35], were found in all the LTRs analyzed. All these elements may account for the observed RE expression. It is to be noted that similar *cis*-elements can be observed in both strands of analyzed LTRs.

### Insertion of new REs in the genome

To investigate whether *Copia *and *Gypsy *transcriptional activity leads to the integration of daughter copies in the genome of *Helianthus annuus*, the IRAP protocol [36] was applied to detect polymorphisms within the HCM line. This sunflower line was subject to eighteen self-pollination cycles, thus it is to be considered as homozygous as indicated by the phenotype uniformity. Since the IRAP protocol displays RE fingerprinting arising from the amplification of neighbour LTRs, new integrated copies of LTR-retroelements can produce polymorphic bands if they insert themselves close enough to a second element, to be amplified by *Taq *DNA polymerase.

**Figure 3:**
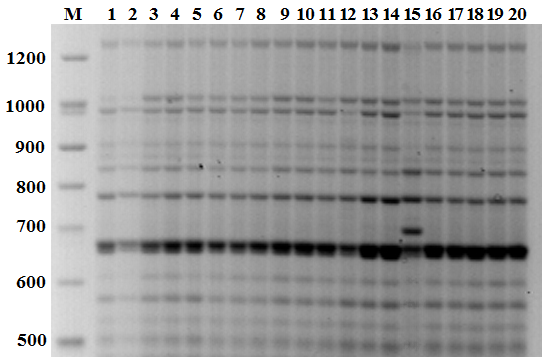
**IRAP analysis of sunflower embryos**. IRAP analysis in 20 sunflower embryos collected in the same flower-head of the highly inbred line HCM. Molecular weight marker (M, Gene Ruler DNA Ladder Mix, Fermentas) was loaded on the left. Embryo # 15 shows a clear polymorphic band.

**Figure 4:**
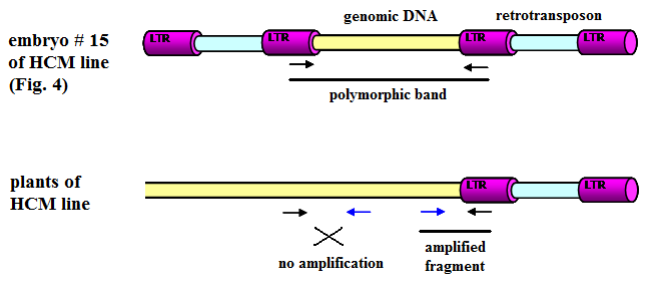
**Schematic representation of polymorphism analysis**. A polymorphic band was detected in the HCM embryo # 15 (see Fig. 4). Forward and reverse primers designed within the inter-retrotransposon locus and directed outward (blue arrows) were coupled with specific LTR primers (black arrows). Only one fragment was amplified from plants of HCM line.

To detect RE-related polymorphisms, primers were designed on the 5'- and the 3'-LTRs ends of *Copia *and *Gypsy *elements. Since RE insertions are mutagenic and the development of plantlets might be aborted, new events of RE integration were surveyed in sunflower embryos from four inflorescences, one for each developmental stage. Sixty-four embryos (7, 14, 21, or 28 days after pollination) were tested, using specific *Copia- *and *Gypsy-*LTR primers, respectively. Only a combination of LTR specific primers (FF C-LTR/C-LTR2), matching a *Copia *retroelement, produced a clear polymorphic band in a 14 day old embryo (Fig. 3). This band was recovered from the gel, and then cloned and sequenced (EMBL acc. number FM209477). The sequence was found to be delimited by the two primers; 3'-*Copia *LTR and 5'-*Copia *LTR ends were present, excluding unspecific primer annealing. The sequence between the two LTRs (665 bp long) was isolated in other HCM plants using primers designed on the inter-retrotransposon sequence and it showed 100% similarity with the same locus related to the polymorphic band. To assess whether *Copia *LTRs flanked this locus in plants of the HCM line, PCRs were performed with primers pointing outward from the genomic locus (Fig. 4). Of the two possible amplified fragments, only one was obtained of the expected molecular weight, containing a portion of 5'- LTR, indicating the occurrence of a *Copia *element in that side of the locus. This suggests that a new *Copia *retrotransposon had inserted itself in the embryo on the other side of the locus.

## Discussion

McClintock [37] addressed the presence and activity of mobile elements within the host as a chance for the genome to cope with challenges to which it is not prepared to react. The genome restructuring action of mobile elements should have made available further genetic variability, increasing the possibility to overcome the challenge.

In the present work, RT-PCR experiments have demonstrated that the *Copia *and the *Gypsy *REs families investigated are transcribed in all *Helianthus annuus *analyzed tissues, i.e. roots, leaves and flowers. Transcripts showed a very low level of non synonymous/synonymous substitution rates. Similar ratios were reported for *Copia*, *Gypsy*, and LINE REs also in other Asteraceae, as *Hieracium aurantiacum*, *Taraxacum officinale *and *Antennaria parlinii*, suggesting a conservative selection [38]. Nine out of ten RE-mRNA transcripts did not show any supernumerary stop codon, supporting the presence of a potentially functional segment of RE pol-proteins. That "parasite" sequences with no apparent function for the host genome tend to maintain their amino acid sequence may be somewhat unexpected. A possible explanation is that only recently inserted elements (i.e., those which have not been subject to mutations yet) are functional.

BLAST screening against EST databases has shown that transcripts related to these REs families are largely transcribed in different *Helianthus *species and in other Asteraceae. Interestingly, G13 and G22 matches are distributed in several species belonging to different genera. It should be noted that, in other dicots, retrotransposon ESTs were reported to find matches only in their host species [18].

The isolation and sequencing of a number of full length *Copia *and *Gypsy *5'-LTRs showed the occurrence of a proper TATA box and putative *cis*-elements in their sequence. Transcripts were isolated using a poly-T primer targeting the 3'-poli-A tail, showing that the 3'-ends of RE transcripts were processed by the host genome. The occurrence of promoter sequences, of functional protein sequences, the RE transcription and the RE-mRNA 3'-end processing are all hints of autonomous retrotransposons.

It is known that REs are subject to inactivation by either mutations or chromatin condensation. Replication allows these elements to survive as genome parasites, but the higher the replication rate the lower will be the host fitness and, consequently, survival of the REs. In the sunflower, although the expression of REs is widespread in all the tissues analyzed, IRAP experiments revealed only one convincing polymorphism, which was attributable to a new integration event. This suggests that, despite a substantial transcriptional activity, RE-mRNAs are quelled and the insertion of new REs is inhibited at a post-transcriptional level, as shown in other species including humans (see [20]).

The amplification of REs in the genome can have some functions for the host. For example, the occurrence in the LTR of putative promoter elements such as those observed in the sunflower could be used by the host to regulate the expression of nearby genes. Constitutively active LTR promoters could determine a housekeeping expression, while tissue-specific LTR promoters would drive the expression of genes in those tissues. An example of gene transcription related to the activity of adjacent retrotransposons was reported in wheat [39]. In mouse oocytes, retrotransposon related transcripts are predominant in the mRNA pool and LTR promoters are responsible for the transcription of a set of genes [40].

The ubiquity of RE transcripts observed in sunflower tissues, and the fact that REs are actively transcribed in standard culture conditions, can support the idea that retrotransposons can be integrated into cell metabolism. For instance, a basal level of retrotransposons transcription would make available the "rough material", namely dsRNAs, that can trigger RE silencing via RNA-directed DNA methylation and chromatin remodeling [41] or via a post-transcriptional mechanism [23]. Double stranded RNA precursors may originate from transcribed nested, head-to-tail oriented LTRs [39], from read-through transcription of two elements which are head-to-tail oriented or from anti-sense strand transcription [42-44].

In this sense, low-copy elements would be the most hazardous for the host, because of the rareness of head-to-tail orientation in the genome, so reducing the efficiency of silencing mechanisms. Accordingly, the few elements in plants for which new insertion events were shown, are three *Copia*-like elements, *Tnt1*, *Tto1*, and *Tos17*, present in a relatively low copy number (< 1,000) per haploid genome (see [45]).

Previous analyses of sunflower REs revealed that they are highly methylated [28]. The families of REs investigated in this study are present in several thousands of copies within the genome and are possibly methylated. However, the widespread transcription of such elements suggests that RE silencing in this species occurs also by degradation of RE mRNAs.

## Conclusion

Retrotransposon transcription was shown in all sunflower tissues analyzed in our experiments. RE activity is not apparently induced by environmental factors or by culture conditions. In one over 64 surveyed embryos a new RE insertion occurred, possibly determining a mutation.

We can speculate that in the sunflower the rarity of insertion events, observed in our experiments despite the consistent transcriptional activity of the *Copia *and *Gypsy *RE families investigated, would be linked to post-transcriptional regulation of REs activity, probably through the degradation of target RE mRNAs.

## Methods

### Plant materials, DNA and RNA extraction

Roots, leaves, embryos, and flowers were collected from plants of the HCM line of *Helianthus annuus*, grown in the field. The HCM line was developed at the Dept. of Crop Plant Biology of University of Pisa after 18 self-pollination cycles, starting from an open-pollinated cultivar, and it is a highly homozygous line, as indicated by phenotype uniformity. Self-pollination was obtained by covering inflorescences to prevent outcrossing. After sampling, tissues were ground in liquid nitrogen. DNA was extracted from embryos and leaves using a CTAB protocol [46] with minor modifications. For total RNA extraction, a MES-guanidine hydrochloride-containing buffer was used following the protocol described by Logeman et al. [47].

### RNA purification

A tuned RNA purification protocol was tailored to avoid genomic DNA contamination, i.e., DNA remnants invalidating RT-PCR analyses. Such a high level of accuracy is crucial especially when analyzing RE expression because of the high frequency of REs in the genomes.

RNA was purified by treatment with DNAse I (Roche). The total amount of DNAse units was set according to the maximum amount of glycerol allowed in the reaction mixture, i.e. 25 μg total RNA, 10 × DNAse I incubation buffer 5 μl, RNAse-free recombinant DNAse I 100 units (10 μl total volume, 5 μl glycerol volume), DEPC water up to 50 μl. Samples were incubated for 1 h at 37°C. After DNAse treatment, RNA purification required a phenol-chloroform extraction and standard precipitation with 1/10 volume 3 M sodium acetate and 2 volumes of cold, 100% ethanol. Using this protocol any possible genomic DNA contamination was prevented [see Additional file 1].

### Expression analyses by RT-PCR

For retrotranscription, total RNA (5 μg) was heated for 3 min at 70°C and retrotranscribed in a 20 μl volume reaction using 400 μM of each deoxynucleotide triphosphate, 0.25 μM poly(T) primer, 1×RT-Buffer, 1 mM DTT, 200 U SuperScript III Reverse Transcriptase (Life Technologies). The same quantity of RNA was processed as above but in the absence of the reverse transcriptase and used as a negative control in RT-PCR.

PCRs were performed using 1/20 volume (1 μl) of retrotranscribed cDNA, 2.5 mM MgCl_2_, 250 μM of each deoxynucleotide triphosphate, 1 μM of each retroelement-specific oligonucleotides (Table 4), 1 U *Taq *DNA polymerase (Biodyne), 20 μl volume reaction. Thermocycling (30 cycles) was performed at 94°C for 30 s, 60°C for 30 s and 72°C for 80 s for G13 RE; at 94°C for 30 s, 55°C for 30 s and 72°C for 80 s for G22 RE; at 94°C for 30 s, 53°C for 30 s and 72°C for 80 s for G30 RE; and at 94°C for 30 s, 57°C for 30 s and 72°C for 30 s for C211 RE. PCR products were visualized on 2% agarose gel and EtBr-stained. Three products for each RE, amplified using RNA isolated from embryos at 28 days after pollination, were cloned in pGEM T-easy vector using manufacturer's instructions, and sequenced.

**Table 4:** Retrotransposon specific primers used in RT-PCR reactions.

RT-PCR primers (*Copia *and *Gypsy*)	
G13F	5'-TCAGACGGATGGGCAGTCTGAGCGA-3'
G13R	5'-ACTCTGGGCCACGACGGGAGTTCCA-3'
G22F	5'-ACGCGTTTGAACTTCAGTACGGCT-3'
G22R	5'-TCAGGACCTCTACGAGCATCC-3'
G30F	5'-GGTCTATCACCGGGTCTCAAC-3'
G30R	5'-TACCCGGAAATAATCGAAGTCGTG-3'
C211F	5'-GCTGGATGTCAGTTCTTAGG-3'
C211R	5'-GATTTCGATGTGTTTGGTCTT-3'

LTR isolation primers (*Copia*, *Gypsy, and universal*)	

*Copia *ChWP3	5'-CGAGATGAGTGCGATGGGTGAAAT-3'
*Gypsy *ChWP4	5'-GCAGAGGTGGGAGATAGTCAGAT-3'
Anch. p	5'-ACCATCGTCCTCAGGTTAGTCAGG-3'
PBS^met^	5'-TAGGTCGGAACAGGCTCTGATACCA-3'
P1FCo	5'-CTGGTGTCTGTAACTTGTCTGTATTCG-3'
P2FCo	5'-AAAGATATGCTTCGATTGATAGACCTC3'
P1FGy	5'-GTGAGTACGTACCAAATTTCGGGAC-3'
P2FGy	5'-TTTCAACTTGGGGATAATGTGACAAC -3'

IRAP primers (*Copia*)	

FA C-LTR i-r	5'-AGAGCATTCTGTCCGAAACAC-3'
FC C-LTR-h	5'-TAGCTTGGATTCCGCACTCG-3'
FF C-LTR	5'-GGTTTAGGTTCGTAATCCTCCGCG-3'
C-LTR r-rt1	5'-CGATAGATGGTCCGAAGGATC-3'
C-LTR 1	5'-AGACACCAGTGGCACCAACA-3'
C-LTR 2	5'-ACAGACACCAGTGGCACCAAC-3'

IRAP primers (*Gypsy*)	

1F G-LTR BO37	5'-GGACAATATCATGGTGCGGTTAC-3'
1F G-LTR DO48	5'-ACCCTTCTTGACGAGACCAGT-3'
G-LTR 1	5'-CTGGTTTTCCTGGGGTGTCA-3'
G-LTR 2	5'-GGGTTGTCACATTATCCCCAAG-3'

Polymorphic band analysis primers (*Copia*)	

FF C-LTR	5'-GGTTTAGGTTCGTAATCCTCCGCG-3'
C-LTR 2	5'-ACAGACACCAGTGGCACCAAC-3'
EMB 1F	5'-TCTTGACATGGGTTGTGGGCT-3'
EMB 1R	5'-TCACATGAACACGGCTCACACA-3'
EMB 2F	5'-AGTCTAATGGGTCAGCATGG-3'
EMB 2R	5'-TCCCTGGTATGAGCCGAAGCTCT-3'

### Sequence analysis

Sequences were aligned using CLUSTAL W [48]. Some adjustments were made by eye. Statistics of sequence polymorphisms were performed using the DnaSP program version 3.51 [49]. Nucleotide diversity (π, i.e. the average number of nucleotide differences per site) and its sampling variance were calculated according to Nei [50], equations 8.4 and 8.12, replacing 2n by n.

Relationships among LTR sequences were investigated by the neighbour-joining (NJ) method (distance algorithm after Kimura), using the PHYLIP program package Version 3.572 [51]: after sequence alignment, 500 versions of the original alignment were generated using the SEQBOOT program; then trees were generated using PROTDIST (or DNADIST) and NEIGHBOR programs, using default options. A strict consensus tree was obtained from the available trees using the CONSENSE program.

### Isolation of Gypsy LTRs

To isolate full length *Gypsy *LTRs a two-step PCR protocol was applied [30]. Firstly, putative partial 3' LTR chromosome walking was performed: specific retrotransposon forward primers designed onto a conserved domain belonging to a *Gypsy Integrase *gene (GenBank Acc. Nr. AJ532592) (*Gypsy *ChWp4, Table 4) were coupled with a random annealing reverse primer (5'-ACCATCGTCCTCAGGTTAGTCAGG-3', Ra A-P). PCR products were amplified using 30 ng DNA, 2.5 mM MgCl_2_, 0.5 μM primers, 1 U *Taq *FirePol (Biodyne) DNA polymerase, 20 μl volume reaction. Thermocycling was performed at 94°C for 30 s, 60°C for 30 s and 72°C for 2 min, for 30 cycles. Products longer than 1,000 bp were cloned and sequenced as above. Clustal analyses were performed to address putative polypurine tract (PPT) whose location is usually a couple of nucleotides before the 3' LTR beginning. Due to the remarkable LTR sequence variability and the lack of large conserved sequence traits, at this stage the 3' boundaries of the 3' LTR within the sequenced clones could not be determined.

In the second step, isolation of complete 5' LTRs was performed. As the retrotransposon LTRs were made identical before the retroelement genome integration, primers designed in the 3'LTR would be expected to match both LTRs. Therefore, specific forward primers were designed downstream of the putative PPT matching *Gypsy*-like 3' LTR, (P1F*Gypsy *and P2F*Gypsy *respectively at bases 51-75 and bases 84-109 after the canonical 5 ' TG) and coupled with a universal primer designed onto the primer binding site (PBS) related to the tRNA^met ^sequence pointing towards the 5' LTR (PBS^met^, 5'-TAGGTCGGAACAGGCTCTGATACCA-3' [52]). Thermocycling was performed at 94°C for 30 s, 57°C for 30 s and 72°C for 60 s, for 30 cycles. PCR products resulting from a semi- nested PCR between P2F*Gypsy*- and PBS-primer were visualized on EtBr-stained agarose gel, cloned as above and sequenced.

### IRAP protocol

Ten primers were designed on *Copia *and *Gypsy *LTR ends (Table 4). Embryos DNAs were amplified using 20 ng DNA, 2.5 mM MgCl_2_, 0.25 μM primers, 1 U *Taq *FirePol (Biodyne) DNA polymerase, 20 μl volume reaction. Thermocycling was performed at 94°C for 30 s, 55°C for 30 s, and 72°C for 150 s. PCR products were visualized on 2% EtBr-stained agarose gel.

### Polymorphic band analysis

A polymorphic fragment was recovered from the gel, cloned in pGEM T-easy vector using the manufacturer's instructions, and sequenced. Specific primers (EMB 1F, EMB 1R, Table 4) were designed to amplify the same genomic locus in individuals of the same HCM line by PCR, using 30 ng DNA, 2.5 mM MgCl_2_, 0.5 μM primers final concentration, 1 U *Taq *FirePol (Biodyne) DNA polymerase, 20 μl volume reaction. Thermocycling was performed at 94°C for 30 s, 60°C for 30 s, 72°C for 2 min. Primers (EMB 2F, EMB 2R, Table 4) were designed on the polymorphic isolated locus. The LTR primers (FF C-LTR, C-LTR2, Table 4) were coupled with EMB 2F and EMB 2R, and PCR was performed using the same reaction mixture as above, at 94°C for 30 s, 57°C for 30 s, 72°C for 1 min. PCR products were cloned as above and sequenced.

## Abbreviations

RE: retrotransposon; TE: transposable element; ORF: open reading frame; LTR: long terminal repeat; IRAP: inter retrotransposon amplified polymorphism.

## Authors' contributions

MV, TG, LN and AC conceived and designed the study. MV performed sequence isolation and identification, expression and phylogenetic analyses, IRAP polymorphism detection. TG contributed to generate the data. MV and AC wrote the manuscript. LN and TG participated in the interpretation and discussion of results and contributed to the writing of the paper. AC is the principal investigator for the *Helianthus *projects and coordinated the study. All authors read and approved the final manuscript.

## Supplementary Material

Additional file 1 Checking DNA contamination in RT-PCR analyses
The file describes the experimental procedures performed to exclude that the results of expression analyses by RT-PCR are altered by possible genomic DNA contamination of cDNA. Click here for file

## References

[B1] Piegu B Guyot R Picault N Roulin A Samiyal A Kim H Collura K Brar DS Jackson S Wing RA Panaud O Doubling genome size without polyploidization: dynamics of retrotransposition driven genomic expansion in *Oryza australiensis*, a wild relative of rice Genome Research 2006 16 1262 1269 10.1101/gr.5290206 16963705 PMC1581435

[B2] Tanskanen JA Sabot F Vicient C Schulman AH Life without GAG: The BARE-2 retrotransposon as a parasite's parasite Gene 2007 390 166 174 10.1016/j.gene.2006.09.009 17107763

[B3] Voytas DF Cummings MO Konieczny A Ausubel FM Rodermel SR *Copia*-like retrotransposons are ubiquitous among plants Proc Natl Acad Sci USA 1992 89 7124 7128 10.1073/pnas.89.15.7124 1379734 PMC49658

[B4] Suoniemi A Tanskanen J Schulman AH *Gypsy*-like retrotransposons are widespread in the plant kingdom Plant Journal 1998 13 699 705 10.1046/j.1365-313X.1998.00071.x 9681012

[B5] Witte CP Le QH Bureau T Kumar A Terminal-repeat retrotransposons in miniature (TRIM) are involved in restructuring plant genomes Proc Natl Acad Sci USA 2001 98 13778 13713 10.1073/pnas.241341898 11717436 PMC61118

[B6] Kalendar R Vicient CM Peleg O Anamthawat-Jonsson K Bolshoy A Schulman AH Large retrotransposon derivatives: abundant, conserved but non autonomous retroelements of barley and related genomes Genetics 2004 166 1437 1450 10.1534/genetics.166.3.1437 15082561 PMC1470764

[B7] Hirochika H Activation of tobacco retrotransposons during tissue culture EMBO Journal 1993 12 2521 2528 8389699 10.1002/j.1460-2075.1993.tb05907.x PMC413490

[B8] Hirochika H Sugimoto K Otsuki Y Tsugawa H Kanda M Retrotransposons of rice involved in mutations induced by tissue culture Proc Natl Acad Sci USA 1996 93 7783 7788 10.1073/pnas.93.15.7783 8755553 PMC38825

[B9] Grandbastien MA Activation of plant retrotransposons under stress conditions Trends Plant Science 1998 3 181 189 10.1016/S1360-1385(98)01232-1

[B10] Grandbastien MA Audeon C Bonnivard E Casacuberta JM Chalhoub B Costa APP Le QH Melayah D Petit M Poncet C Tam SM Van Sluys MA Mhiri C Stress activation and genomic impact of Tnt1 retrotransposons in Solanaceae Cytogenet Genome Res 2005 110 229 241 10.1159/000084957 16093677

[B11] Brunner S Fengler K Morgante M Tingey S Rafalski A Evolution of DNA sequence nonhomologies among maize inbreds Plant Cell 2005 17 343 360 10.1105/tpc.104.025627 15659640 PMC548811

[B12] Scherrer B Isidore E Klein P Kim JS Bellec A Chalhoub B Keller B Feuillet Large intraspecific haplotype variability at the Rph7 locus results from rapid and recent divergence in the barley genome Plant Cell 2005 17 361 374 10.1105/tpc.104.028225 15659632 PMC548812

[B13] Kalendar R Tanskanen J Immonen S Nevo E Schulman AH Genome evolution of wild barley (*Hordeum spontaneum*) by BARE-1 retrotransposons dynamic in response to sharp microclimatic divergence Proc Natl Acad Sci USA 2000 12 6603 6607 10.1073/pnas.110587497 PMC18673 10823912

[B14] Kobayashi S Goto-Yamamoto N Hirochika H Retrotransposon-induced mutations in grape skin color Science 2004 304 982 10.1126/science.1095011 15143274

[B15] Clark RM Wagler TN Quijada P Doebley J A distant upstream enhancer at the maize domestication gene *tb1 *has pleiotropic effects on plant and inflorescence architecture Nat Genet 2006 38 594 597 10.1038/ng1784 16642024

[B16] Morgante M De Paoli E Radovic S Transposable elements and the plant pan-genomes Curr Opin Plant Biology 2007 10 149 155 10.1016/j.pbi.2007.02.001 17300983

[B17] Meyers BC Tingey SV Morgante M Abundance, distribution, and transcriptional activity of repetitive elements in the maize genome Genome Research 2001 11 1660 1676 10.1101/gr.188201 11591643 PMC311155

[B18] Vicient CM Jaaskelainen M Kalendar R Schulman AH Active retrotransposon are a common feature of grass genomes Plant Physiology 2001 125 1283 1292 10.1104/pp.125.3.1283 11244109 PMC65608

[B19] Vicient CM Schulman AH *Copia*-like retrotransposons in rice genome: few and assorted Genome Letters 2002 1 35 47 10.1166/gl.2002.002

[B20] Slotkin RK Martienssen R Transposable elements and the epigenetic regulation of the genome Nat Rev Genet 2007 8 272 285 10.1038/nrg2072 17363976

[B21] Volpe A Kidner C Hall IM Teng G Grewal SIS Martienssen RA Regulation of heterochromatic silencing and histone H3 Lysine-9 methylation by RNAi Science 2002 297 1833 1837 10.1126/science.1074973 12193640

[B22] Vagin VV Sigova A Li C Seitz H Gvozdev V Zamore PD A distinct small RNA pathway silences selfish genetic elements in the germline Science 2006 313 320 324 10.1126/science.1129333 16809489

[B23] Lisch D Epigenetic regulation of transposable elements in plants Annu Rev Plant Biol 2009 60 43 66 10.1146/annurev.arplant.59.032607.092744 19007329

[B24] Ungerer MC Strakosh SC Zhen S Zhen Y Genome expansion in three hybrid sunflower species is associated with retrotransposon proliferation Current Biology 2006 16 872 873 10.1016/j.cub.2006.09.020 17055967

[B25] Ungerer MC Strakosh SC Stimpson KM Proliferation of Ty3/*Gypsy*-like retrotransposons in hybrid sunflower taxa inferred from phylogenetic data BMC Biology 2009 7 40 doi:10.1186/1741-7007-7-40 19594956 10.1186/1741-7007-7-40 PMC2715380

[B26] Cavallini A Zolfino C Natali L Cionini G Cionini PG Nuclear DNA changes within *Helianthus annuus *L.: origin and control mechanism Theor Appl Genet 1986 73 20 26 10.1007/BF00273713 24232467

[B27] Santini S Cavallini A Natali L Minelli S Maggini F Cionini PG *Ty1/Copia*- and *Ty3/Gypsy-like *DNA sequences in *Helianthus *species Chromosoma 2002 111 192 200 10.1007/s00412-002-0196-2 12355209

[B28] Cavallini A Natali L Zuccolo A Giordani T Jurman I Ferrillo V Vitacolonna N Sarri V Cattonaro F Ceccarelli M Cionini PG Morgante M Analysis of transposons and repeat composition of the sunflower (*Helianthus annuus *L.) genome Theor Appl Genet 2009 doi 10.1007/s00122-009-1170-7 10.1007/s00122-009-1170-7 19826774

[B29] Schilling EE Phylogenetic analysis of *Helianthus *(Asteraceae) based on chloroplast DNA restriction site data Theor Appl Genet 1997 94 925 933 10.1007/s001220050497

[B30] Vukich M Schulman AH Giordani T Natali L Kalendar R Cavallini A Genetic variability in sunflower (*Helianthus annuus *L.) and in the *Helianthus *genus as assessed by retrotransposon-based molecular markers Theor Appl Genet 2009 DOI: 10.1007/s00122-009-1106-2 19618160 10.1007/s00122-009-1106-2

[B31] Higo K Ugawa Y Iwamoto M Korenaga T Plant *cis*-acting regulatory DNA elements (PLACE) database: 1999 Nucleic Acids Res 1999 27 297 300 10.1093/nar/27.1.297 9847208 PMC148163

[B32] Yanagisawa S Schmidt RJ Diversity and similarity among recognition sequences of Dof transcription factors Plant J 1999 17 209 214 10.1046/j.1365-313X.1999.00363.x 10074718

[B33] Gowik U Burscheidt J Akyildiz M Schlue U Koczor M Streubel M Westhoff P *Cis*-Regulatory elements for mesophyll-specific gene expression in the C4 plant *Flaveria trinervia*, the promoter of the C4 phosphoenolpyruvate carboxylase gene Plant Cell 2004 16 1077 1090 10.1105/tpc.019729 15100398 PMC423201

[B34] Terzaghi WB Cashmore AR Light-regulated transcription Annu Rev Plant Physiol Plant Mol Biol 1995 46 445 474 10.1146/annurev.pp.46.060195.002305

[B35] Allen RD Bernier F Lessard PA Beachy RN Nuclear factors interact with a soybean beta-conglycinin enhancer Plant Cell 1989 1 623 631 10.1105/tpc.1.6.623 2535514 PMC159797

[B36] Kalendar R Schulman AH IRAP and REMAP for retrotransposon-based genotyping and fingerprinting Nature Protocols 2006 1 2478 2484 10.1038/nprot.2006.377 17406494

[B37] McClintock B The significance of responses of the genome to challenge Science 1984 226 792 801 10.1126/science.15739260 15739260

[B38] Docking TR Saadé FE Elliot MC Shoen DJ Retrotransposon sequence variation in four asexual plant species J Mol Evol 2006 62 375 387 10.1007/s00239-004-0350-y 16547645

[B39] Kashkush K Feldman M Levy AA Transcriptional activation of retrotransposons alters the expression of adjacent genes in wheat Nature Genetics 2003 33 102 106 10.1038/ng1063 12483211

[B40] Peaston AE Evsikov AV Graber JH De Vries WN Holbrook AE Solter D Knowles BB Retrotransposons regulate host genes in mouse oocytes and preimplantation embryos Dev Cell 2004 7 597 606 10.1016/j.devcel.2004.09.004 15469847

[B41] Huettel B Kanno T Daxinger L Bucher E Winden J Van Der Matzke AJM Matzke M RNA-directed DNA methylation mediated by DRD1 and PolIVb: a versatile pathway for transcriptional gene silencing in plants Biochim Biophys Acta 2007 1769 358 374 17449119 10.1016/j.bbaexp.2007.03.001

[B42] Copeland SC Mann VH Brindley PJ Both sense and antisense strands of the LTR of the *Schistosoma mansoni Pao*-like retrotransposon *Sinbad *drive luciferase expression Mol Genet Genomics 2007 277 161 170 10.1007/s00438-006-0181-1 17131159

[B43] Belo A Nobuta K Venu RC Janardhanan PE Wang GL Meyers BC Transposable element regulation in rice and *Arabidopsis*: diverse patterns of active expression and siRNA-mediated silencing Tropical Plant Biol 2008 1 72 84 10.1007/s12042-007-9008-4

[B44] Shpiz S Kwon D Rozovsky Y Kalmykova rasiRNA pathway controls antisense expression of *Drosophila *telomeric retrotransposons in the nucleus Nucl Acids Res 2009 37 268 278 10.1093/nar/gkn960 19036789 PMC2615633

[B45] Yamazaki M Tsugawa H Miyao A Yano M Wu J Yamamoto S Matsumoto T Sasaki T Hirochika H The rice retrotransposon *Tos17 *prefers low-copy-number sequences as integration targets Mol Genet Genomics 2001 265 336 344 10.1007/s004380000421 11361345

[B46] Doyle JJ Doyle JL Isolation of plant DNA from fresh tissue Focus 1989 12 13 15

[B47] Logemann J Schell J Willmitzer L Improved method for the isolation of RNA from plant-tissue Analytical Biochemistry 1987 163 16 20 10.1016/0003-2697(87)90086-8 2441623

[B48] Thompson JD Desmond G Gibson H Gibson TJ CLUSTAL W improving the sensitivity of progressive multiple sequence alignment through sequence weighting, position-specific gap penalties and weight matrix choice Nucleic Acids Res 1994 22 4673 4680 10.1093/nar/22.22.4673 7984417 PMC308517

[B49] Rozas J Rozas R DnaSP version 3: an integrated program for molecular population genetics and molecular evolution analysis Bioinformatics 1999 15 174 175 10.1093/bioinformatics/15.2.174 10089204

[B50] Nei M Molecular Evolutionary Genetics 1987 Columbia University Press, New York, NY

[B51] Felsenstein J PHYLIP: phylogeny inference package Cladistics 1989 5 164 166

[B52] Kalendar R Tanskanen J Chang W Antonius K Sela H Peleg O Schulman AH Cassandra retrotransposons carry independently transcribed 5S RNA Proc Natl Acad Sci USA 2008 105 5833 5838 10.1073/pnas.0709698105 18408163 PMC2311327

