# Supplementary material for: Copia and Gypsy retrotransposons activity in sunflower (Helianthus annuus L.)
Source: BMC Plant Biol. 2009 Dec 23;9:150. doi: 10.1186/1471-2229-9-150 (PMC2805666; doi:10.1186/1471-2229-9-150)
Supplement: Additional file 1 — Checking DNA contamination in RT-PCR analyses The file describes the experimental procedures performed to exclude that the results of expression analyses by RT-PCR are altered by possible genomic DNA contamination of cDNA. [file 1471-2229-9-150-S1.PDF]

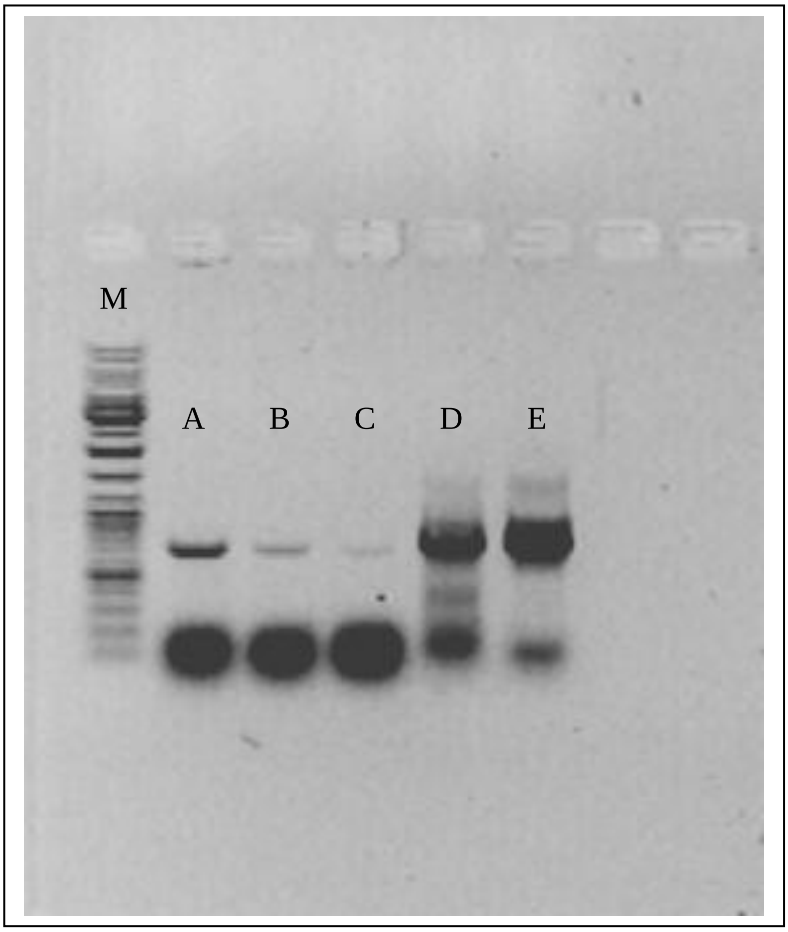

Additional Material: Checking DNA contamination in RT-PCR analyses

A major problem affecting RT-PCR analysis of repeated DNA sequences is the contamination of isolated RNA by genomic DNA. The occurrence of genomic DNA can be verified performing simple PCRs on non-retrotranscribed RNA treated with DNAse. We tested scalar concentrations of DNAse and performed PCR using primers specific to the G13 retroelement. Three aliquots of total RNA were purified respectively with 2, 4, or 8 µl of DNAse. 1µl of DNAse-treated RNA (non retrotranscribed) was used as template in control PCR reactions (lane A, 2 µl of DNAse; lane B, 4 µl; lane C, 8 µl). In each sample, primers amplified the target sequence indicating genomic DNA contaminating remnants. On the contrary, no amplification signal was observed using 10 µl of DNAse (see Figure 1).

In lane D, the result of a PCR reaction using the same primers and 20ng of genomic DNA as template is reported: the difference between D and A indicates that the DNA contamination in isolated RNA is very slight. In lane E, the result of an RT-PCR reaction is reported, in which 1µl of purified (with 2 µl of DNAse, as in A) and retrotranscribed RNA was used; here a large band is observed, the difference between E and A should be attributed to amplification from cDNA, confirming that the G13 retroelement is transcribed.

It is to be noted that in the same RT-PCR conditions, actin primers did not yield any band (not shown), indicating that when analysing transcription of repeated elements specific primers are required to test DNA contamination in control PCR.

M, molecular marker (100 bp ladder). 2% agarose gel stained with ethidium bromide.
